# Reliability Modeling for Humidity Sensors Subject to Multiple Dependent Competing Failure Processes with Self-Recovery

**DOI:** 10.3390/s18082714

**Published:** 2018-08-18

**Authors:** Jia Qi, Zhen Zhou, Chenchen Niu, Chunyu Wang, Juan Wu

**Affiliations:** 1 School of Measurement and Communication Engineering, Harbin University of Science and Technology, Harbin 150080, China; qjia89@hrbust.edu.cn (J.Q.); samueland@126.com (C.N.); wangchunyu230281@163.com (C.W.); 2 College of Precision Instruments and Opto-electronics Engineering, Tianjin University, Tianjin 300072, China; taozi_xixi@163.com

**Keywords:** reliability model, humidity sensor, self-recovery, dependent competing failure, random shocks

## Abstract

Recent developments in humidity sensors have heightened the need for reliability. Seeing as many products such as humidity sensors experience multiple dependent competing failure processes (MDCFPs) with self-recovery, this paper proposes a new general reliability model. Previous research into MDCFPs has primarily focused on the processes of degradation and random shocks, which are appropriate for most products. However, the existing reliability models for MDCFPs cannot fully characterize the failure processes of products such as humidity sensors with significant self-recovery, leading to an underestimation of reliability. In this paper, the effect of self-recovery on degradation was analyzed using a conditional probability. A reliability model for soft failure with self-recovery was obtained. Then, combined with the model of hard failure due to random shocks, a general reliability model with self-recovery was established. Finally, reliability tests of the humidity sensors were presented to verify the proposed reliability model. Reliability modeling for products subject to MDCFPs with considering self-recovery can provide a better understanding of the mechanism of failure and offer an alternative method to predict the reliability of products.

## 1. Introduction

Humidity sensors have been widely used in scientific research and industry applications, such as in the quality control of integrated circuit manufacturing, biological products and pharmaceuticals, and the control of chemical and physical processes [1,2,3,4,5,6,7]. The breakdown of humidity sensors may cause the failure of control, detection, and display functions of a system. The rigorous working environment and the diversification of structure and function have put forward increasingly strong reliability requirements for humidity sensors.

The reliability of humidity sensors as an important performance parameter represents the ability of humidity sensors to work without failure under the stated conditions for a specified period. Reliability is a long-term quality indicator for products and cannot be detected before leaving the factory. Reliability modeling is an important tool to evaluate the reliability of products. Reliability modeling for products that experience only soft or hard failure has been extensively explored in the previous studies. Hard failure is when the product’s performance remains unchanged before failure and the product suddenly fails at a certain time [8,9,10,11,12], whereas soft failure is the continuous degradation process of a product’s performance. When a product’s performance exceeds a certain value, soft failure occurs [13,14,15]. However, due to the complexity of the internal structure and working environment, humidity sensors may deteriorate due to corrosion, fatigue, wear, and other causes. Humidity sensors may also break down suddenly through external shocks. These failure processes compete against each other, and whichever occurs first will cause the humidity sensor to fail. In this case, it is difficult to characterize the failure processes of humidity sensors accurately and comprehensively using soft failure or hard failure alone, which may lead to inaccuracies in the reliability design, analysis, and evaluation. Reliability modeling for humidity sensors and many other products is in line with the actual failure process by combining soft failure and hard failure. Therefore, the reliability theory of competing failure should be used to model the reliability of humidity sensors and many other products.

Competing failure can be categorized into independent and dependent competing failure. In practical applications, competing failure processes are generally dependent on each other. Simply describing the relationship between different failure processes independently often produces an over-estimation of the product’s reliability or may even result in unnecessary loss due to untimely maintenance [16,17,18,19,20]. For products subject to multiple dependent competing failure processes (MDCFPs), Peng et al. [21] assumed that some shocks were fatal, which could cause a product’s hard failure. Most shocks had little effect on the performance of the product, which could cause sudden damage to continuous performance degradation. In [21], sudden damages were accumulative. Rafiee et al. [22] analyzed a maintenance policy for products subject to MDCFPs and classified random shocks in accordance with the effect of shocks on the failure of products, in which fatal shocks caused hard failure, and non-fatal shocks caused instantaneous damage on degradation. An and Sun [23] discussed a maintenance policy of products subject to MDCFPs and proposed that not all non-fatal shocks caused sudden damages. Only when the amplitudes of shocks were higher than a certain threshold could the shock cause damages. A similar assumption was also found in [24]. Huynh et al. [25] modeled the degradation process through a stochastic process where the degradation process was shown to be strictly increasing. Liu et al. [26] developed a maintenance policy for systems subject to MDCFPs and assumed that the degradation process was an incremental process when system uptime was within a cycle.

Previous literature in this area has some limitations in terms of the reliability research of MDCFPs. Most researchers assumed that sudden damages were accumulative, and that the degradation process strictly increased. This means that previous reliability studies into MDCFPs ignored self-recovery, which is not appropriate for some products. For example, the drift of humidity sensors may undergo a reversible process. External shocks such as rapid humidity increases may cause positive offsets in the long-term continuous drift of humidity sensors. When returning to mild humidity conditions, the offsets slowly decrease. This self-recovery phenomenon exists in many other products and materials, such as mechanics, electronics, micro-electro mechanical system, and self-reconfigurable robotics [27]. After a careful literature review, we found that Liu et al. [28] considered self-recovery and proposed many ideal assumptions regarding self-recovery like the self-recovery process was linear. However, Liu et al. did not develop a specific reliability model with self-recovery. The question of how to characterize the effect of self-recovery reasonably is a challenge that needs to be solved in the reliability modeling for products subject to MDCFPs, and in the reliability analysis of humidity sensors.

In this paper, we developed a new general reliability model for humidity sensors subject to MDCFPs by considering self-recovery. We investigated both hard and soft failure processes. Hard failure is caused by random shocks, whereas soft failure is characterized by a random coefficient regression (RCR) model with positive increments. The RCR model is used to characterize the long-term continuous drift process of humidity sensors, which is caused by physical aging. The positive increments are sudden offsets caused by random shocks. In particular, we took into account that not all non-fatal shocks could cause offsets to the long-term continuous drift. When the inter-arrival time of two continuous shocks is sufficiently large, offsets may decrease. Only when the inter-arrival time of two continuous shocks is under a certain temporal threshold is there a chance that offsets remain. After this, the generality of the developed model is discussed. By setting different parameters, the model can be transformed into different reliability models. Finally, reliability tests of the humidity sensors are given to illustrate the model.

The remainder of this paper is arranged as follows. Section 2 lists the assumptions used in the reliability modeling studies in accordance with the humidity sensors failure process. In Section 3, a reliability model is developed for humidity sensors subject to MDCFPs by considering self-recovery. Moreover, we discuss the generality of the developed model and transform the model into four different reliability models by setting different parameters. In Section 4, reliability tests of the humidity sensors are presented to verify our model. Then, the effects of the parameters on the reliability model are discussed. Section 5 summarizes this paper with concluding remarks.

## 2. Description of Humidity Sensors Failure Process

**Figure 1:**
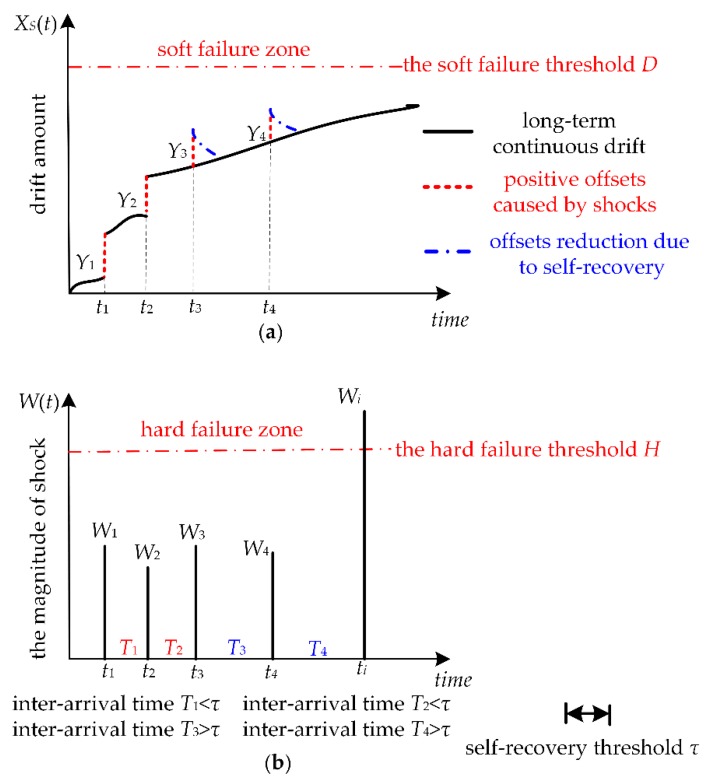
MDCFPs of humidity sensors. (**a**) Soft failure process of humidity sensors; (**b**) Hard failure process of humidity sensors.

The failure of humidity sensors is due to two dependent competing failure processes as shown in Figure 1. The soft failure process is shown in Figure 1a, which is determined by long-term continuous drift, random shocks, and self-recovery. The long-term continuous drift between the humidity sensor’s measured values and the actual humidity is caused by physical aging and is often affected by random shocks. When the shock amplitude is less than a certain constant value *H*, that is, a non-fatal shock, the shock may cause an additional positive offset that arises between the sensor measured value and the actual humidity. The phenomenon wherein shocks can cause increases to degradation exists in many products. In particular, we propose that not all non-fatal shocks cause additional positive offsets. Only frequent shocks with an inter-arrival time of two continuous shocks less than a certain threshold can cause an increase to the long-term continuous drift of humidity sensors. This means that frequent shocks can cause positive offsets, and these offsets are accumulative. If the inter-arrival time of two continuous shocks is larger than a threshold value, once humidity sensors are returned to mild conditions, the offsets may decrease slowly. The self-recovery process is one of decline in positive offsets after a temperature or humidity shock. Self-recovery may be observed once samples are returned to mild environmental conditions. This indicates that this positive drift is a temporary offset that is fully reversible with slow kinetics after returning the sensor to a mild environment. The positive offset is not due to the irreversible damage to the sensing polymer, such as the hydrolysis of the chemical bonds linking the monomers. The positive offset is caused by the rapid environmental change which may self-recover. The hard failure process is shown in Figure 1b. The humidity sensor’s exposure to extreme shocks that exceed the threshold level *H* may cause hard failure. In summary, we assumed that humidity sensors are subject to MDCFPs which include both soft and hard failure. The two competing failure processes are dependent due to the shared exposure to random shocks.

Any of the following conditions cause humidity sensors to fail: (1) the drift of the humidity sensors are beyond the soft failure threshold *D*, or (2) the magnitude of any shock exceeds the threshold level *H*.

The specific assumptions used in the reliability modeling in accordance with the humidity sensors failure process can be summarized as follows. The notation used in formulating the reliability models is cited in the Appendix A.

Soft failure occurs when the total drift of the humidity sensors is beyond the failure threshold *D*. The total drift amount includes the long-term continuous drift with time, positive offsets caused by random shocks, and offset reduction due to self-recovery.

When any shock amplitude exceeds the threshold level *H*, hard failure occurs.

Shocks occur by a homogeneous Poisson process (HPP), and the rate of HPP is *λ*. The magnitude of the *i-*th shock load is denoted as *W_i_* for *i* = 1, 2, …, ∞. *W_i_*is normally distributed *W_i_*~*N*(*μ_W_*, *σ_W_*).

When the inter-arrival time of two continuous shocks is greater than a certain threshold, positive offsets caused by the shocks may decrease, otherwise, positive offsets may remain.

The number of shocks is independent of the magnitude of the shock loads and the positive offsets caused by shocks.

## 3. Reliability Modeling for Humidity Sensors with Considering Self-Recovery

### 3.1. Reliability Modeling for Humidity Sensors Subject to Soft Failure

Figure 1a demonstrates that the soft failure of humidity sensors occurs when the total drift exceeds *D*. The total drift *X_S_*(*t*) includes long-term continuous drift, positive offsets, and offset reduction due to self-recovery. The long-term continuous drift is due to ageing, *X*(*t*), is given as

*X*(*t*) = *a* + *βt* (1)

The *X*(*t*) may follow a linear degradation path with random coefficients or a randomized logistic degradation path. Furthermore, it may be necessary to apply a transformation to result in a linear form [29,30]. For illustration purposes, we used a linear degradation path to characterize the long-term continuous drift, where the parameter *β* is a random variable that corresponds to normal distribution *β*~*N*(*μ*, *σ*^2^) and where *a* is a constant.

We assumed that the positive offsets caused by shocks are normally distributed, and denoted as *Y_i_* for *i* = 0, 1, 2, …, *∞*, and *Y_i_*~*N*(*μ_Y_*, *σ_Y_*). When considering self-recovery, the positive offsets distribution function is

(2) $$P({\overset{⌢}{Y}}_{i}<y)=P(Y_{i}<y,T_{i}\leq \tau )+P(Y_{i}<y,T_{i}>\tau )=(1-e^{-\lambda \tau })\Phi (y)$$

where Φ(•) is the cumulative density function (CDF) of a standard normally distributed variable. *t_i_* represents the arrival time of the *i-*th shock, and *T_i_* represents the inter-arrival time between the *i-*th shock and the (*i* + 1)-th shock.

The cumulative positive offset *S*(*t*) is given by a compound Poisson process

(3) $$S(t)=\{\begin{array}{cc} \sum_{i=0}^{N(t)}{\hat{Y}}_{i} & N(t)>0\\ 0 & N(t)=0 \end{array}$$

where *N*(*t*) is the number of random shocks.

Ignoring self-recovery, the cumulative positive offset *S*_1_(*t*) can be calculated as

(4) $$S_{1}(t)=\{\begin{array}{c} \sum_{i=0}^{N(t)}Y_{i}\begin{array}{cc} & N(t)>0 \end{array}\\ 0\begin{array}{cc} & N(t)=0 \end{array} \end{array}$$

The difference of the cumulative positive offset between considering and ignoring self-recovery can be calculated as

$$S_{1}(t)-S(t)=\sum_{i=0}^{N(t)}Y_{i}-(1-e^{-\lambda \tau })Y_{i}=\sum_{i=0}^{N(t)}e^{-\lambda \tau }Y_{i}\;N\left(t\right)>0$$

The larger the number of shocks, the higher the shock frequency and the better self-recovery performance, the greater the difference of whether it considers self-recovery.

The probability of the *i-*th shock occurring by time *t* is

(5) $$P\{N(t)=i\}=\frac{{\left(\lambda t\right)}^{i}}{i!}e^{-\lambda t}$$

Furthermore, if we consider *G*(*t*) to be the CDF of ${\hat{Y}}_{i}$ at time *t*, and *G^j^*(*t*) a *j* convolution of *G*(*t*), then the CDF of the *S*(*t*) can be derived as

(6) $$P\{S(t)\leq x\}=P\{\sum_{i=0}^{N(t)}{\hat{Y}}_{i}\leq x\}=\sum_{j=0}^{N(t)}P\{\sum_{i=0}^{N(t)}{\hat{Y}}_{i}\leq x|N(t)=j\}P\{N(t)=j\}=\sum_{j=0}^{N(t)}G^{j}(x)\frac{{\left(\lambda t\right)}^{j}e^{-\lambda t}}{j!}$$

The total drift *X_S_*(*t*) of humidity sensors can be expressed as

*X_S_*(*t*) = *X*(*t*) + *S*(*t*) (7)

By using Equations (1), (3) and (6), the reliability model of humidity sensors subject to soft failure can be derived as

(8) $$F_{x}(t)=P\{X(t)+S(t)<D\}=P\{X(t)+\sum_{i=0}^{N(t)}{\hat{Y}}_{i}<D\}=\sum_{i=0}^{N(t)}P\{(X(t)+\sum_{i=0}^{N(t)}{\hat{Y}}_{i}<D)|N(t)=j\}\times P\{N(t)=j\}$$

The reliability model in Equation (8) can be derived for a specific case with a normally distributed *Y_i_* and *β*

(9) $$R(t)=\Phi (\frac{D-\mu t}{\sigma t})e^{-\lambda t}+\sum_{i=1}^{N(t)}\Phi (\frac{D-\mu t-i(1-e^{-\lambda \tau })\mu_{Y}}{\sqrt{i{\left(1-e^{-\lambda \tau }\right)}^{2}\sigma_{Y}{}^{2}+\sigma^{2}}t^{2}})\cdot \frac{{\left(\lambda t\right)}^{i}e^{-\lambda t}}{i!}=\sum_{i=0}^{N(t)}\Phi (\frac{D-\mu t-i(1-e^{-\lambda \tau })\mu_{Y}}{\sqrt{i{\left(1-e^{-\lambda \tau }\right)}^{2}\sigma_{Y}{}^{2}+\sigma^{2}t^{2}}})\cdot \frac{{\left(\lambda t\right)}^{i}e^{-\lambda t}}{i!}$$

### 3.2. Reliability Modeling for Humidity Sensors Subject to Random Shocks

When the shock load exceeds the threshold level *H*, hard failure occurs. According to the stress-strength model [31], the probability of surviving the *i*-th shock is shown as

(10) $$P(W_{i}<H)=F_{W}(H)\;i=1,\;2,\;\ldots ,\;N(t).$$

In this paper, a stochastic extreme shock model was used to characterize the random shocks that cause hard failure. The magnitude of the *i-*th shock is denoted as *W_i_* for *i* = 1, 2, …, *N*(*t*), and *W_i_*~ *N*(*μ_W_*, *σ_W_*). Therefore, the probability of survival in Equation (10) is

(11) $$F_{W}(H)=P(W_{i}<H)=\Phi (\frac{H-\mu_{W}}{\sigma_{W}})\;i=1,\;2,\;\ldots ,\;N(t).$$

where the Φ(•) is the CDF of a standard normally distributed variable.

### 3.3. Reliability Modeling for Humidity Sensors Subject to MDCFPs

Hard or soft failure can cause humidity sensors to fail. The reliability function can be derived as 

(12) $$\begin{array}{l} R(t)=P(X(t)<D,N(t)=0)+\sum_{i=1}^{N(t)}P(W_{1}<H,\ldots ,W_{N(t)}<H,X(t)+\sum_{i=1}^{N(t)}{\hat{Y}}_{i}<D,N(t)=i)\\ =P(X(t)<D,N(t)=0)+\sum_{i=1}^{N(t)}F_{W}{\left(H\right)}^{i}P(X(t)+\sum_{i=1}^{N(t)}{\hat{Y}}_{i}<D)|N(t)=i)\times P\{N(t)=i\} \end{array}$$

The reliability function can be expressed for a more specific case

(13) $$\begin{array}{l} R(t)=P(X(t)<D,N(t)=0)+\sum_{i=1}^{N(t)}P(W_{1}<H,\ldots ,W_{N(t)}<H,X(t)+\sum_{i=1}^{N(t)}{\hat{Y}}_{i}<D,N(t)=i)\\ =\Phi (\frac{D-\mu t}{\sigma t})e^{-\lambda t}+\sum_{i=1}^{N(t)}\Phi (\frac{D-\mu t-i(1-e^{-\lambda \tau })\mu_{Y}}{\sqrt{\sigma^{2}t^{2}+i{\left(1-e^{-\lambda \tau }\right)}^{2}\sigma_{Y}^{2}}})\cdot \frac{{\left(\lambda t\right)}^{i}e^{-\lambda t}}{i!}\cdot {\left[\Phi (\frac{H-\mu_{W}}{\sigma_{W}})\right]}^{i}\\ =\sum_{i=0}^{N(t)}\Phi (\frac{D-\mu t-i(1-e^{-\lambda \tau })\mu_{Y}}{\sqrt{\sigma^{2}t^{2}+i{\left(1-e^{-\lambda \tau }\right)}^{2}\sigma_{Y}^{2}}})\cdot \frac{{\left(\lambda t\right)}^{i}e^{-\lambda t}}{i!}\cdot {\left[\Phi (\frac{H-\mu_{W}}{\sigma_{W}})\right]}^{i} \end{array}$$

As shown in Equation (13), when 0 < *τ* < ∞, the smaller the value of *τ*, the stronger the product’s self-recovery. When *τ*_1_ < *τ*_2_, offsets caused by shocks with an inter-arrival time greater than *τ*_1_ can recover, which includes the offsets caused by shocks with the inter-arrival time between *τ*_1_ and *τ*_2_. Therefore, the smaller the value of *τ*, the more the offsets recover, the less the degradation volume, and the higher the reliability.

Based on Equation (13), the probability density function (PDF) of the failure time is

(14) $$\begin{array}{l} f(t)=-\frac{dR(t)}{dt}=-\sum_{i=1}^{N(t)}{\left[\Phi (\frac{H-\mu_{W}}{\sigma_{W}})\right]}^{i}\phi (\frac{D-\mu t-i(1-e^{-\lambda \tau })\mu_{Y}}{\sqrt{\sigma^{2}t^{2}+i(1-e^{-\lambda \tau })\sigma_{Y}{}^{2}}})\\ \times (\frac{-\mu (\sigma^{2}t^{2}+i(1-e^{-\lambda \tau })\sigma_{Y}{}^{2})-\sigma^{2}t(D-\mu t-i(1-e^{-\lambda \tau })\mu_{Y})}{{\left(\sigma^{2}t^{2}+i(1-e^{-\lambda \tau })\sigma_{Y}{}^{2}\right)}^{\frac{3}{2}}})\times \frac{{\left(\lambda t\right)}^{i}e^{-\lambda t}}{i!}\\ -\sum_{i=1}^{N(t)}{\left[\Phi (\frac{H-\mu_{W}}{\sigma_{W}})\right]}^{i}\Phi (\frac{D-\mu t-i(1-e^{-\lambda \tau })\mu_{Y}}{\sqrt{\sigma^{2}t^{2}+i(1-e^{-\lambda \tau })\sigma_{Y}{}^{2}}})\times \frac{\lambda {\left(\lambda t\right)}^{i-1}e^{-\lambda t}(-\lambda t+i)}{i!}\\ -\phi (\frac{D-\mu t}{\sigma t})\times (-\frac{D}{\sigma t^{2}})e^{-\lambda t}+\lambda \Phi (\frac{D-\mu t}{\sigma t})e^{-\lambda t} \end{array}$$

where *φ*(•) is the PDF of a standard normally distributed variable.

### 3.4. Some Special Cases

With different parameters, the reliability model Equation (13) can be transformed into different reliability models and coincides with models with a slight difference to the previous literature.

When *τ* = ∞, the reliability model Equation (13) can be transformed into a reliability model for dependent competing failure as shown in Equation (15). The model of Equation (15) ignores self-recovery as with the previous literature [21]. As *τ* = ∞ means that when the inter-arrival time of two continuous shocks is smaller than infinite, a shock can cause positive offsets to the continuous long-term drift, that is, all shocks can cause offsets. 

Ignoring self-recovery (*τ* = ∞), the reliability is shown as

(15) $$\begin{array}{l} R(t)=P(X(t)<D,N(t)=0)+\sum_{i=1}^{N(t)}P(W_{1}<H,\ldots ,W_{N(t)}<H,X(t)+\sum_{i=1}^{N(t)}Y_{i}<D,N(t)=i)\\ =\Phi (\frac{D-\mu t}{\sigma t})e^{-\lambda t}+\sum_{i=1}^{N(t)}\Phi (\frac{D-\mu t-i\mu_{Y}}{\sqrt{\sigma^{2}t^{2}+i\sigma_{Y}^{2}}})\cdot \frac{{\left(\lambda t\right)}^{i}e^{-\lambda t}}{i!}\cdot {\left[\Phi (\frac{H-\mu_{W}}{\sigma_{W}})\right]}^{i}\\ =\sum_{i=0}^{N(t)}\Phi (\frac{D-\mu t-i\mu_{Y}}{\sqrt{\sigma^{2}t^{2}+i\sigma_{Y}^{2}}})\cdot \frac{{\left(\lambda t\right)}^{i}e^{-\lambda t}}{i!}\cdot {\left[\Phi (\frac{H-\mu_{W}}{\sigma_{W}})\right]}^{i} \end{array}$$

Based on Equation (15), the PDF of the failure time is derived as

(16) $$\begin{array}{l} f(t)=-\frac{dR(t)}{dt}=-\sum_{i=1}^{N(t)}\left[\Phi (\frac{H-\mu_{W}}{\sigma_{W}}\right){]}^{i}\phi (\frac{D-\mu t-i\mu_{Y}}{\sqrt{\sigma^{2}t^{2}+i\sigma_{Y}{}^{2}}})\times (\frac{-\mu (\sigma^{2}t^{2}+i\sigma_{Y}{}^{2})-\sigma^{2}t(D-\mu t-i\mu_{Y})}{{\left(\sigma^{2}t^{2}+i\sigma_{Y}{}^{2}\right)}^{\frac{3}{2}}})\\ \times \frac{{\left(\lambda t\right)}^{i}e^{-\lambda t}}{i!}-\sum_{i=1}^{N(t)}\left[\Phi (\frac{H-\mu_{W}}{\sigma_{W}}\right){]}^{i}\Phi (\frac{D-\mu t-i\mu_{Y}}{\sqrt{\sigma^{2}t^{2}+i\sigma_{Y}{}^{2}}})\times \frac{\lambda {\left(\lambda t\right)}^{i-1}e^{-\lambda t}(-\lambda t+i)}{i!}-\phi (\frac{D-\mu t}{\sigma t})\times (-\frac{D}{\sigma t^{2}})e^{-\lambda t}\\ +\lambda \Phi (\frac{D-\mu t}{\sigma t})e^{-\lambda t} \end{array}$$

When *τ* = 0, the reliability model of Equation (13) is transformed into a reliability model for independent competing failure as shown in Equation (17). As *τ* = 0 means that positive offsets can recover when the inter-arrival time of two continuous shocks is greater than 0, that means all offsets can recover. It also means that shocks do not cause offsets to long-term continuous drift when *τ* = 0, that is, hard failure and soft failure are independent of each other. This reliability model is similar to the model used in the previous study [32].

When soft failure and hard failure are independent (*τ* = 0), the reliability is shown as

(17) $$\begin{array}{l} R(t)=P(X(t)<D,N(t)=0)+\sum_{i=1}^{N(t)}P(W_{1}<H,\ldots ,W_{N(t)}<H,X(t)<D,N(t)=i)\\ =\Phi (\frac{D-\mu t}{\sigma t})e^{-\lambda t}+\sum_{i=1}^{N(t)}\Phi (\frac{D-\mu t}{\sigma t})\cdot \frac{{\left(\lambda t\right)}^{i}e^{-\lambda t}}{i!}\cdot {\left[\Phi (\frac{H-\mu_{W}}{\sigma_{W}})\right]}^{i}=\sum_{i=0}^{N(t)}\Phi (\frac{D-\mu t}{\sigma t})\cdot \frac{{\left(\lambda t\right)}^{i}e^{-\lambda t}}{i!}\cdot {\left[\Phi (\frac{H-\mu_{W}}{\sigma_{W}})\right]}^{i} \end{array}$$

Based on Equation (17), the PDF of the failure time is derived as

(18) $$\begin{array}{l} f(t)=-\frac{dR(t)}{dt}=-\sum_{i=0}^{N(t)}\left[\Phi (\frac{H-\mu_{W}}{\sigma_{W}}\right){]}^{i}\times \phi (\frac{D-\mu t}{\sigma t})\times (-\frac{D}{\sigma t^{2}})\frac{{\left(\lambda t\right)}^{i}e^{-\lambda t}}{i!}\\ -\sum_{i=0}^{N(t)}\left[\Phi (\frac{H-\mu_{W}}{\sigma_{W}}\right){]}^{i}\times \Phi (\frac{D-\mu t}{\sigma t})\times \frac{\lambda {\left(\lambda t\right)}^{i-1}e^{-\lambda t}(-\lambda t+i)}{i!} \end{array}$$

Reliability modeling for products that experience soft failure only concerns the performance degradation process. By setting the parameters of random shock in Equation (13) to 0 and ignoring hard failure, the reliability model of Equation (13) can be converted to the reliability model based on performance degradation.

(19) $$R(t)=P(X(t)<D)=\Phi (\frac{D-\mu t}{\sigma t})$$

Based on Equation (19), the PDF of the failure time is derived as

(20) $$f(t)=\phi (\frac{D-\mu t}{\sigma t})(-\frac{D}{\sigma t^{2}})$$

Traditional reliability theory only focuses on hard failure. By setting the parameters of soft failure in Equation (13) to 0, the reliability model of Equation (13) can be converted to the traditional reliability model, which only considers hard failure due to random shocks.

(21) $$R(t)=\sum_{i=0}^{N(t)}{\left[\Phi (\frac{H-\mu_{W}}{\sigma_{W}})\right]}^{i}\frac{{\left(\lambda t\right)}^{i}e^{-\lambda t}}{i!}$$

Based on Equation (21), the PDF of the failure time is derived as

(22) $$f(t)=\sum_{i=1}^{N(t)}{\left[\Phi (\frac{H-\mu_{W}}{\sigma_{W}})\right]}^{i}\times \frac{\lambda {\left(\lambda t\right)}^{i-1}e^{-\lambda t}(-\lambda t+i)}{i!}-\lambda e^{-\lambda t}$$

The reliability model Equation (13) developed in this paper can be transformed into different reliability models seen in previous literature, as shown in Table 1. This means that models 1, 2, 3, and 4 are special cases of the reliability model developed in this paper.

## 4. Numerical Examples and Results

The following two examples in this section are presented to illustrate the model discussed in the previous section.

### 4.1. Example I

A humidity sensors reliability test conducted at AMS Netherlands BV Laboratories was used here to verify the proposed model [33]. Ageing of the sensor may cause a measured value long-term drift. This long-term drift is a continuous degradation process and is often affected by random shocks. When subject to random shocks, such as a rapid increase in humidity, positive offsets may be caused to the long-term continuous drift, especially when humidity sensors return to mild humidity conditions for a long time, that is, if the inter-arrival time of two continuous shocks is long enough, positive offsets will slowly decrease. In contrast, if the inter-arrival times are less than a certain value, positive offsets may remain.

To illustrate the model developed in this paper, we set the parameters shown in Table 2. The reliability model in this paper was based on the statistical analysis of a pseudo failure life and model of hard failure due to random shocks. The pseudo failure life was obtained by extrapolating the degradation path. In the linear degradation path *X*(*t*) = *a* + *βt*, the distribution of *β* can be obtained by recording the drift data of the humidity sensor. It was assumed that *β* is a normally distributed random variable, that is, the degradation volume at any time *t* following a normal distribution. We assumed that the humidity sensors did not degenerate at the initial time, that is, *a* = 0. From the test results, we also obtained the soft failure threshold *D* and the hard failure threshold *H*. We assumed the size of the shock loads as *W_i_* following a distribution, and consequently the positive offset *Y_i_* also followed a normal distribution.

**Figure 2:**
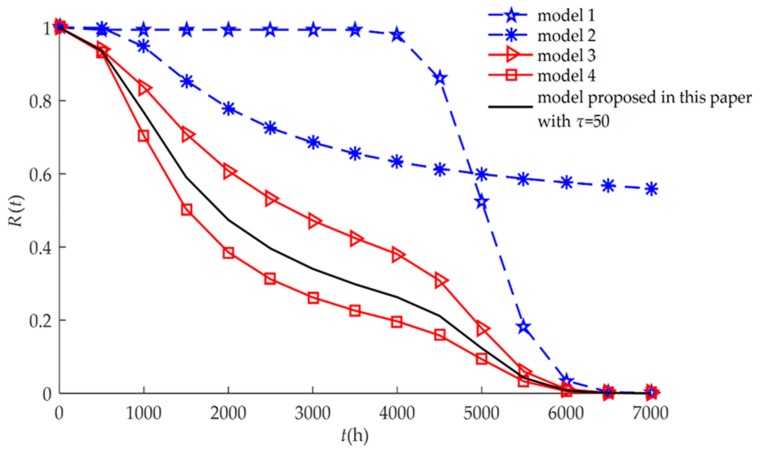
Comparison of *R*(*t*) for different models for example I.

The four different reliability models used in previous studies are special cases of the proposed model as discussed in Section 3.4. All models are drawn and compared in Figure 2. We found that when considering only hard or soft failure (Models 1 and 2), the reliabilities were higher than when both failures had a competitive relationship (Models 3, 4, and the proposed model). For three reliability models of competing failure, Models 3 and 4 were special cases of the proposed model. The reliabilities of the competing failure processes had a similar trend of change. Between 1000 h and 5000 h, the reliability of the independent competing failure (Model 3) was higher than the reliability of the dependent competing failure with self-recovery (the proposed model). When it was assumed that the relationship between soft failure and hard failure was independent, random shock did not affect the long-term continuous drift of the humidity sensors. The degradation amount of the independent competing failure was less than the degradation amount of the dependent competing failure with self-recovery. If we assumed that the failure processes were independent, then the computed reliability might be higher than the actual reliability of the humidity sensors. At the same time, the degradation amount of the dependent competing failure with self-recovery (the proposed model) was less than the degradation amount of the dependent competing failure without considering self-recovery (Model 4). If we ignored the self-recovery processes, then the computed reliability might be smaller than the actual reliability of the humidity sensors. Therefore, for the reliability modeling of some products such as humidity sensors, the self-recovery processes need to be considered.

**Figure 3:**
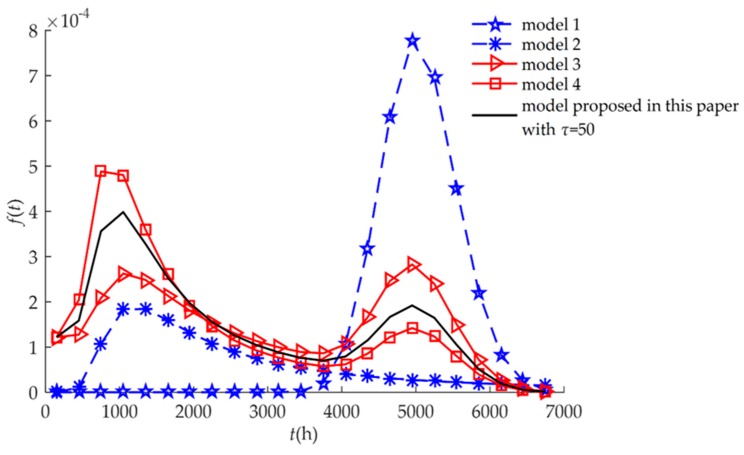
Comparison of *f*(*t*) for different models for example I.

The failure rate functions of Equations (14), (16), (18), (20), and (22) are shown in Figure 3. When only hard failure was considered (Model 1), the failure rate increased significantly after 4000 h. In contrast, when only soft failure was considered (Model 2), the rate was mainly concentrated prior to 4000 h. For the three competing models (Models 3, 4, and the proposed model), the failure rate functions were almost non-zero throughout the service life of the humidity sensors, that is, the humidity sensors could fail at any time during service. When only soft failure or hard failure was considered, the humidity sensors could fail within a specified time.

**Figure 4:**
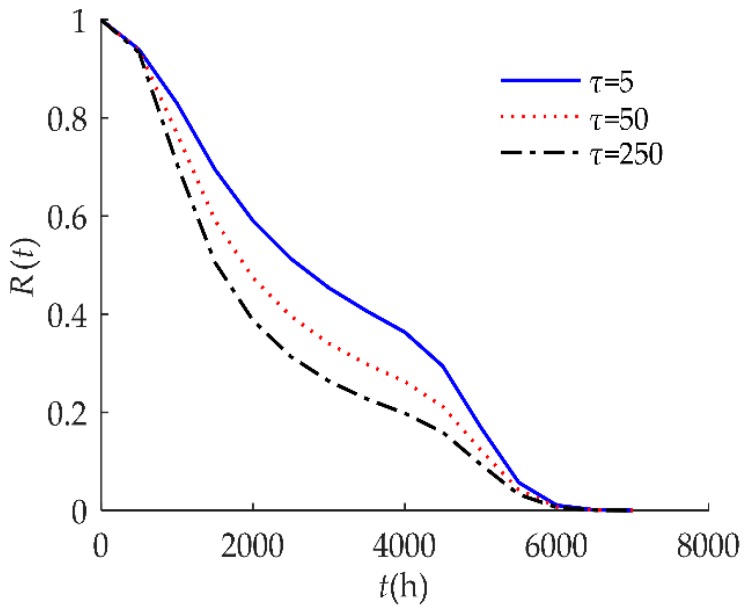
Sensitivity analysis of *R*(*t*) on *τ* for example I.

**Figure 5:**
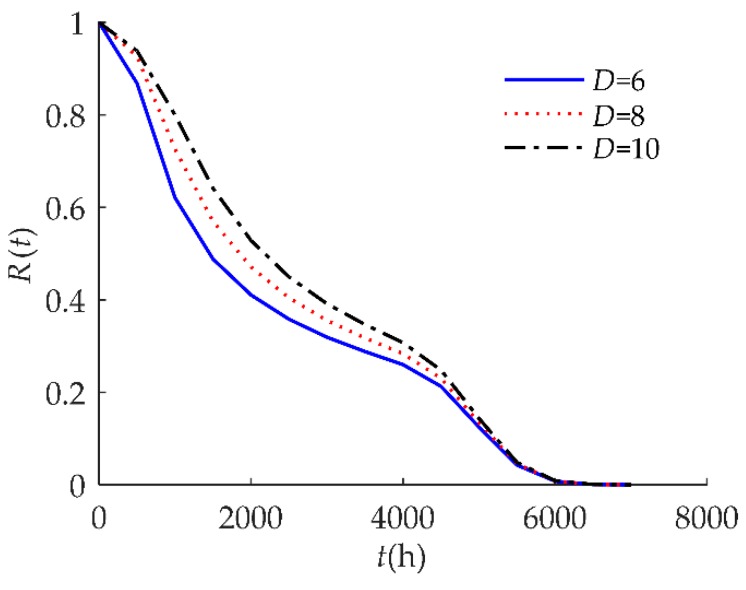
Sensitivity analysis of *R*(*t*) on *D* for example I.

**Figure 6:**
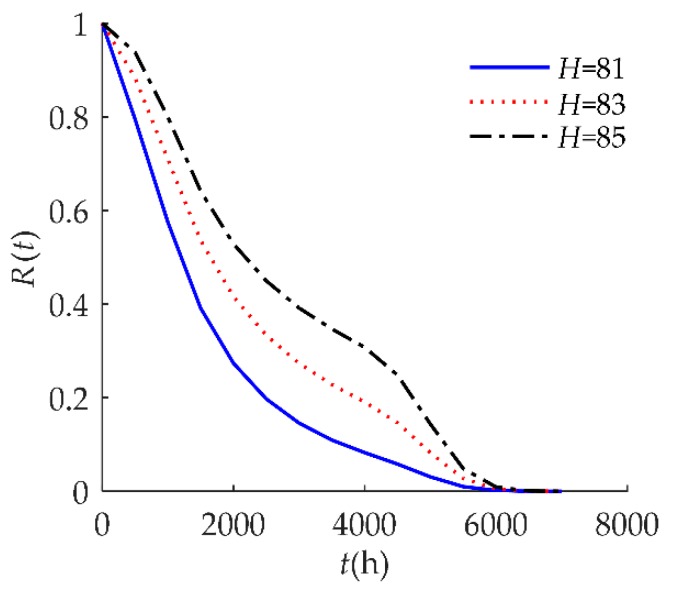
Sensitivity analysis of *R*(*t*) on *H* for example I.

**Figure 7:**
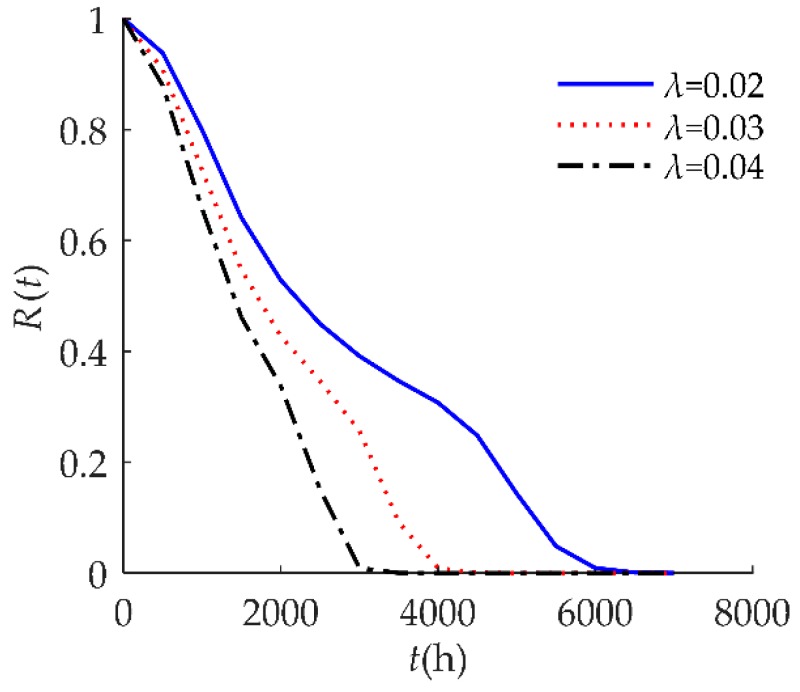
Sensitivity analysis of *R*(*t*) on *λ* for example I.

To explore the influence of parameters on the reliability of humidity sensors, sensitivity analyses of *R*(*t*) on *τ*, *D*, *H*, *λ* are presented in Figure 4, Figure 5, Figure 6 and Figure 7 respectively for example I.

Figure 4 indicates that the self-recovery threshold *τ* had a significant effect on *R*(*t*). When *τ* decreased, the reliability of the humidity sensor increased after 1000 h. As discussed in Section 3.3, the smaller the value of *τ*, the higher the reliability of the products.

Figure 5 indicates that *R*(*t*) as sensitive to the soft failure threshold *D*. When *D* decreased from 10 to 6, *R*(*t*) decreased, which means that the reliability is lower when *D* gets smaller.

In Figure 6, the hard failure threshold *H* had an obvious effect on *R*(*t*). When the hard failure threshold *H* decreased, *R*(*t*) decreased. An explanation for this may be that products with a higher *H* have a better ability to resist shock.

In Figure 7, we observed that *R*(*t*) was susceptible to the random shock rate *λ*. When *λ* decreased, *R*(*t*) shifted to the right. This result indicates that a larger *λ* decreases reliability performance. An explanation for this might be that the higher the shock rate, the more positive offsets on the degradation value, so failure occurs at a much earlier time.

### 4.2. Example II

A case study of a solid state relative humidity (RH) sensor reliability analysis by the University of Wisconsin-Madison is provided to illustrate the model [34]. The drift of capacitance–RH characteristic is the dominant failure mode for a solid state RH sensor at 85 °C/85% RH. In the temperature test of a solid state RH sensor, the sensor breaks down when the magnitude of the temperature shock is above a certain level. In addition, positive offsets to the drift of the capacitance–RH characteristic are caused when the temperature shock is non-fatal. In particular, when the humidity sensors return to mild conditions for a long time, the positive offsets slowly decrease. To illustrate the model developed in this paper, we set the parameters shown in Table 3. The linear degradation path was *X*(*t*) = *a* + *βt*, where *a* = 0 and *β* is normally distributed was obtained by the test data. The shock size and positive offsets caused by the shocks were assumed to be normally distributed.

**Figure 8:**
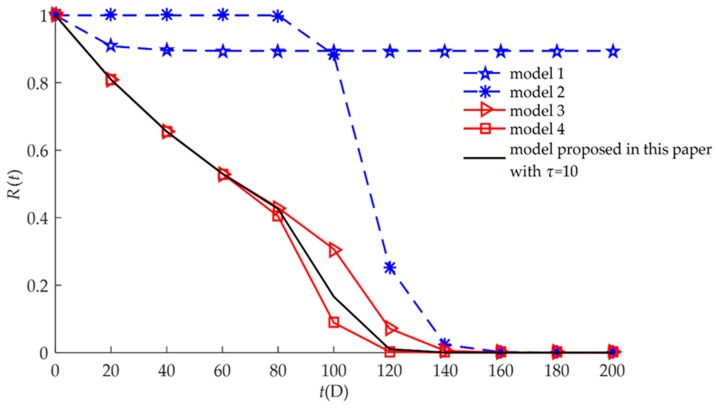
Comparison of *R*(*t*) for different models for example II.

**Figure 9:**
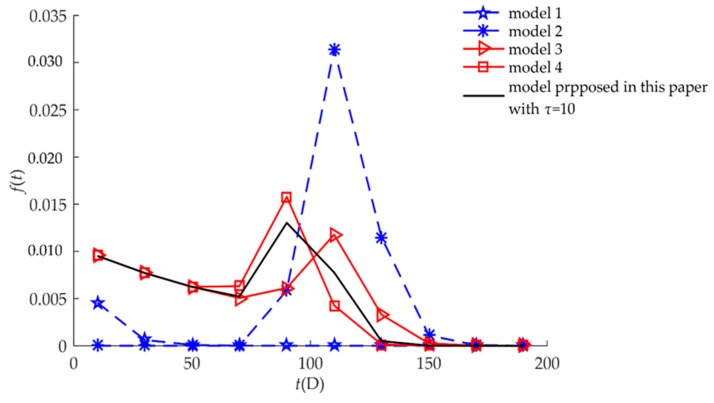
Comparison of *f*(*t*) for different models for example II.

The four different reliability models (Models 1, 2, 3, and 4) used in previous studies are special cases of the proposed model as discussed in Section 3.4. All models are drawn and compared in Figure 8. The corresponding failure rate functions are shown in Figure 9. We also found that when considering only hard or soft failure, the reliabilities were higher than that when both failures had a competitive relationship, the failure rate functions were not 0 within a certain time, and not all the whole time of service. The three reliabilities of competing failure had some of the same change trends. The reliability of the independent competing is the highest, followed by the reliability of the dependent competing with considering self-recovery.

**Figure 10:**
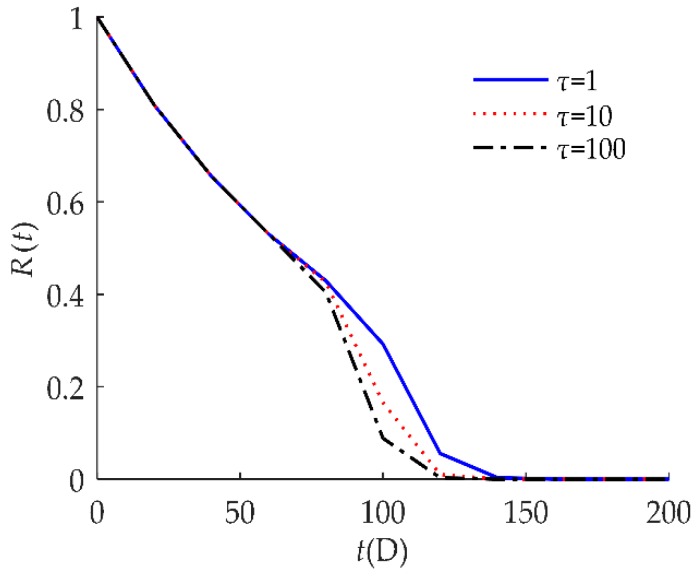
Sensitivity analysis of *R*(*t*) on *τ* for example II.

**Figure 11:**
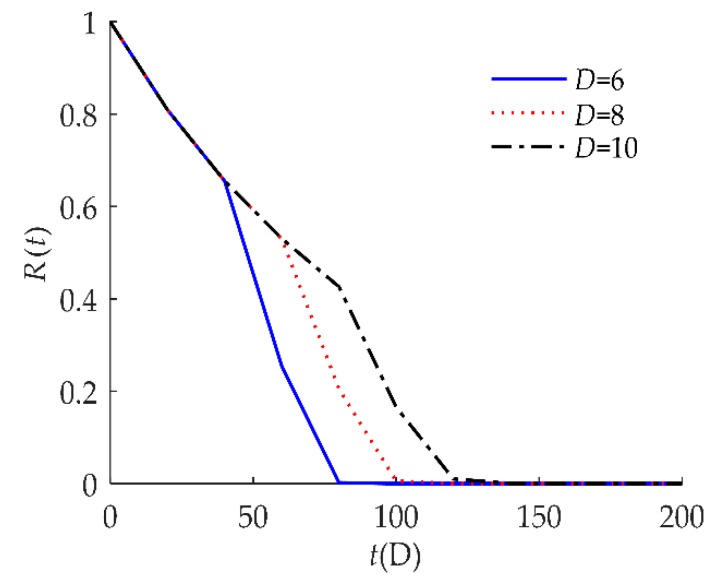
Sensitivity analysis of *R*(*t*) on *D* for example II.

**Figure 12:**
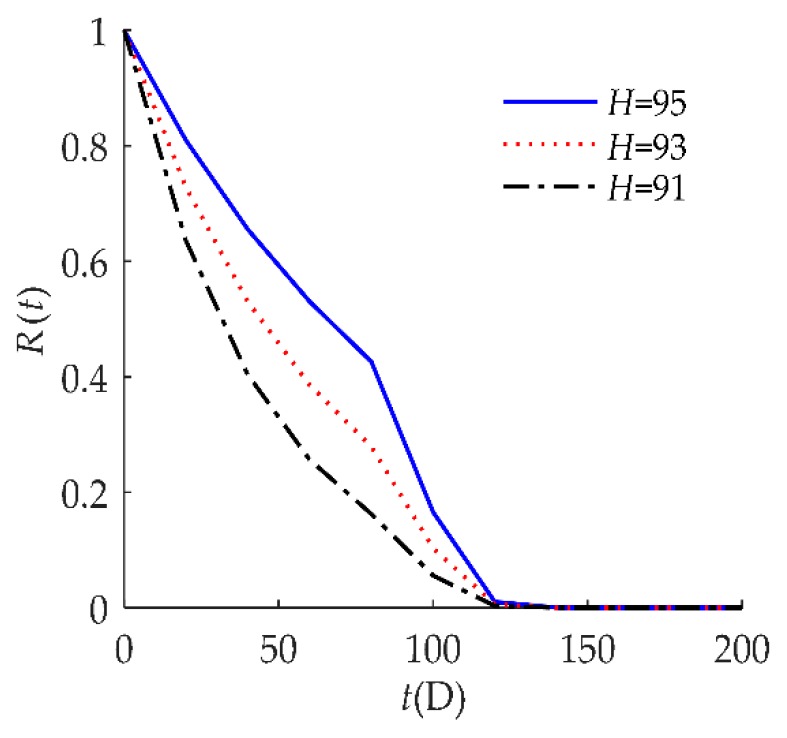
Sensitivity analysis of *R*(*t*) on *H* for example II.

**Figure 13:**
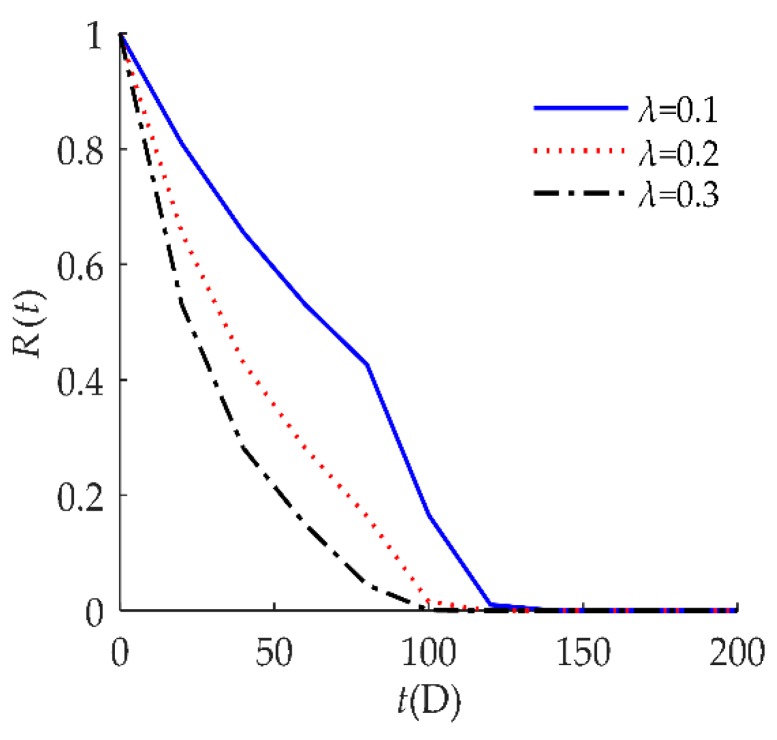
Sensitivity analysis of *R*(*t*) on *λ* for example II.

To explore the influence of the parameters on the reliability of humidity sensors, the sensitivity analyses of *R*(*t*) on *τ*, *D*, *H*, and *λ* are presented in Figure 10, Figure 11, Figure 12 and Figure 13, respectively for example II. These indicate that the reliability performance was better for a smaller self-recovery threshold *τ*, larger soft failure threshold *D*, larger hard failure threshold *H*, or smaller random shock rate *λ*.

## 5. Conclusions

In this paper, we proposed a new and more general reliability model for humidity sensors subjected to dependent competing failure with considering self-recovery. This paper analyzed the condition of self-recovery, that is, it focused on the effect of the inter-arrival time of shocks on continuous degradation. On this basis, a reliability model for soft failure that considered self-recovery was established. Combined with the reliability analysis of hard failure due to shocks, a new reliability model for dependent competing failure that considered self-recovery was developed. By adjusting the different parameters, the generality of the developed model was discussed. It was found that the four different reliability models used in previous studies were the special cases of the model developed in this paper. This new model represents a major extension on previous studies. We presented examples to demonstrate the reliability model and analyzed the effects of the parameters on reliability. For further studies, additional terms of the self-recovery condition can be considered, such as the magnitude of the shocks.

## Figures and Tables

**Table 1:** Reliability models.

Model	Description of the Failure Process	Expression
Model 1	This model characterizes hard failure which is caused by a stochastic shock process.	Equation (21)
Model 2	This model characterizes soft failure process. Products may not be subject to random shocks.	Equation (19)
Model 3	This model characterizes independent competing failure processes. Soft failure and hard failure are independent.	Equation (17)
Model 4	This model characterizes MDCFPs but ignores self-recovery. All non-fatal shocks can cause sudden increases in degradation.	Equation (15)
Model proposed in this paper	This model characterizes MDCFPs by considering self-recovery and can be transformed into four different reliability models (Models 1, 2, 3, and 4) by varying parameters.	Equation (13)

**Table 2:** Parameters of the reliability model for example I.

Parameter	Value	Description
*D*	10	The soft failure threshold is 10: when the drift amount rises by 10%, soft failure occurs.
*H*	85	The hard failure threshold is 85: when relative humidity exceeds 85%, hard failure occurs.
*β*	*N*(0.0005, 0.005^2^)	The drift rate.
*α*	*α* = 0	The drift value at the initial time (*t* = 0).
*W_i_*	*N*(65, 8^2^)	The *i-*th shock amplitude.
*λ*	0.02/h	The rate of a homogeneous Poisson random shock process.
*Y_i_*	*N*(0.2, 0.02^2^)	The positive offsets caused by the *i-*th shock.

**Table 3:** Parameters of the reliability model for example II.

Parameter	Value	Description
*D*	10	The soft failure threshold is 10: when the drift amount rises by 10%, soft failure occurs.
*H*	95	The hard failure threshold is 95: when ambient temperature exceeds 95 °C, hard failure occurs.
*β*	N(0.0893, (0.0090)^2^)	The drift rate of capacitance–RH characteristic at 85 °C/85% RH.
*α*	*α* = 0	The drift value at the initial time (*t* = 0).
*W_i_*	*N*(85, 8^2^)	The *i-*th shock amplitude.
*λ*	0.1/Day	The rate of a homogeneous Poisson random shock process.
*Y_i_*	*N*(0.2, 0.02^2^)	The positive offsets caused by the *i-*th shock.

## Appendix A

The notation used in formulating the reliability models is now listed.

*t*	time
*X*(*t*)	drift of the measured value compared to reference caused by physical ageing at time *t*
*N*(*t*)	the number of shocks
*Y_i_*	a positive offset between the sensor measured value and the actual conditions caused by the *i**-*th shock
*W_i_*	the magnitude of the *i**-*th shock
*D*	the threshold of soft failure
*H*	the threshold of hard failure
*λ*	the rate of a homogeneous Poisson random shock process
*X_S_(t)*	total drift volume at time *t* composed to long-term continuous drift, positive offsets caused by random shocks, and offset reduction due to self-recovery
*R*(*t*)	time-dependent reliability
*T_i_*	inter-arrival time between the *i**-*th shock and the (*i* + 1)*-*th shock
*τ*	the self-recovery threshold
*S(t)*	cumulative positive offset caused by random shocks at time *t*

## References

[1] Hernandezrivera D., Rodriguezroldan G., Moramartinez R., Suastegomez E. (2017). A Capacitive Humidity Sensor Based on an Electrospun PVDF/Graphene Membrane. Sensors.

[2] Dessler A.E., Sherwood S.C. (2009). A Matter of Humidity. Science.

[3] Liehr S., Breithaupt M., Krebber K. (2017). Distributed Humidity Sensing in PMMA Optical Fibers at 500 nm and 650 nm Wavelengths. Sensors.

[4] Previati M., Canone D., Bevilacqua I., Boetto G., Pognant D., Ferraris S. (2012). Evaluation of wood degradation for timber check dams using time domain reflectometry water content measurements. Ecol. Eng..

[5] Boudaden J., Steinmabl M., Endres H.E., Drost A., Eisele I., Kutter C., Muller-Buschbaum P. (2018). Polyimide-Based Capacitive Humidity Sensor. Sensors.

[6] Park H., Lee S., Jeong S.H., Jung U.H., Park H., Lee M.G., Kim S., Lee J. (2018). Enhanced Moisture-Reactive Hydrophilic-PTFE-Based Flexible Humidity Sensor for Real-Time Monitoring. Sensors.

[7] Blank T.A., Eksperiandova L.P., Belikov K.N. (2016). Recent trends of ceramic humidity sensors development: A review. Sens. Actuators B Chem..

[8] Zuo M.J., Jiang R., Yam R.C.M. (2002). Approaches for reliability modeling of continuous-state devices. IEEE Trans. Reliab..

[9] Barnett T.S., Grady M., Purdy K., Singh A.D. (2005). Exploiting defect clustering for yield and reliability prediction. IEE Proc.-Comput. Dig. Tech..

[10] Huang W., Askin R.G. (2004). A generalized SSI reliability model considering stochastic loading and strength aging degradation. IEEE Trans. Reliab..

[11] Mallor F., Santos J. (2003). Classification of Shock Models in System Reliability.

[12] Fan J., Ghurye S.G., Levine R.A. (2000). Multicomponent Lifetime Distributions in the Presence of Ageing. J. Appl. Probab..

[13] Lu C.J., Meeker W.Q. (1993). Using Degradation Measures to Estimate a Time-to-Failure Distribution. Technometrics.

[14] Kharoufer J.P., Cox S.M. (2005). Stochastic models for degradation-based reliability. IIE Trans..

[15] Park C., Padgett W.J. (2006). Stochastic degradation models with several accelerating variables. IEEE Trans. Reliab..

[16] Chien Y.H., Shen S.H., Zhang Z.G., Love E. (2006). An extended optimal replacement model of systems subject to shocks. Eur. J. Oper. Res..

[17] Keedy E., Feng Q. (2012). A physics-of-failure-based reliability and maintenance modeling framework for stent deployment and operation. Reliab. Eng. Syst. Saf..

[18] Li W., Pham H. (2005). An inspection-maintenance model for systems with multiple competing processes. IEEE Trans. Reliab..

[19] Cha J.H., Pulcini G. (2016). A Dependent Competing Risks Model for Technological Units Subject to Degradation Phenomena and Catastrophic Failures. Qual. Reliab. Eng. Int..

[20] Wang Y., Pham H. (2011). A Multi-Objective Optimization of Imperfect Preventive Maintenance Policy for Dependent Competing Risk Systems with Hidden Failure. IEEE Trans. Reliab..

[21] Peng H., Feng Q., Coit D.W. (2010). Reliability and maintenance modeling for systems subject to multiple dependent competing failure processes. IIE Trans..

[22] Rafiee K., Feng Q., Coit D.W. (2015). Condition-based maintenance for repairable deteriorating systems subject to generalized mixed shock model. IEEE Trans. Reliab..

[23] An Z., Sun D. (2017). Reliability modeling for systems subject to multiple dependent competing failure processes with shock loads above a certain level. Reliab. Eng. Syst. Saf..

[24] Rafiee K., Feng Q., Coit D.W. (2017). Reliability assessment of competing risks with generalized mixed shock models. Reliab. Eng. Syst. Saf..

[25] Huynh K.T., Barros A., Berenguer C., Castro I.T. (2011). A periodic inspection and replacement policy for systems subject to competing failure modes due to degradation and traumatic events. Reliab. Eng. Syst. Saf..

[26] Liu X., Li J., Alkhalifa K.N., Hamouda A.S., Coit D.W., Elsayed E.A. (2013). Condition-based maintenance for continuously monitored degrading systems with multiple failure modes. IIE Trans..

[27] Regina F., Richard M.C., Benjamin D., Alan P., Asutosh T., Giovanna D.M.S. (2013). Self-healing and self-repairing technologies. Int. J. Adv. Manuf. Technol..

[28] Liu H., Yeh R.H., Cai B. (2017). Reliability modeling for dependent competing failure processes of damage self-healing systems. Comput. Ind. Eng..

[29] Wang Y., Pham H. (2011). Imperfect preventive maintenance policies for two-process cumulative damage model of degradation and random shocks. Int. J. Syst. Assur. Eng. Manag..

[30] Bae S.J., Kou W., Kvam P.H. (2007). Degradation models and implied lifetime distribution. Reliab. Eng. Syst. Saf..

[31] Rafiee K., Feng Q., Coit D.W. (2014). Reliability modeling for dependent competing failure processes with changing degradation rate. IIE Trans..

[32] Wang Z., Huang H., Li Y., Xiao N. (2011). An approach to reliability assessment under degradation and shock process. IEEE Trans. Reliab..

[33] Jose S., Vooge F., Schaar C.V.D., Nath S., Nenadovic N., Vanhelmont F., Lous E.J., Suy H., Zandt M.I., Sakic A. Reliability tests for modeling of relative humidity sensor drifts. Proceedings of the 2017 IEEE Reliability Physics Symposium.

[34] Denton D.D., Jaafar M.A.S., Ralston A.R.K. The long term reliability of a switched-capacitor relative humidity sensor system. Proceedings of the IEEE International Symposium on Circuits and Systems.

